# Recent Advances on the Innate Immune Response to *Coxiella burnetii*

**DOI:** 10.3389/fcimb.2021.754455

**Published:** 2021-11-02

**Authors:** Guido Sireci, Giusto Davide Badami, Diana Di Liberto, Valeria Blanda, Francesca Grippi, Laura Di Paola, Annalisa Guercio, José de la Fuente, Alessandra Torina

**Affiliations:** ^1^ Central Laboratory of Advanced Diagnostic and Biological Research (CLADIBIOR), Department of Biomedicine, Neurosciences and Advanced Diagnostics (BIND), University Hospital “Paolo Giaccone”, Università degli studi di Palermo, Palermo, Italy; ^2^ Istituto Zooprofilattico Sperimentale della Sicilia, Palermo, Italy; ^3^ SaBio Health and Biotechnology, Instituto de Investigación en Recursos Cinegéticos, IREC -Spanish National Research Council CSIC - University of Castilla-La Mancha UCLM - Regional Government of Castilla-La Mancha JCCM, Ciudad Real, Spain; ^4^ Department of Veterinary Pathobiology, Center for Veterinary Health Sciences, Oklahoma State University, Stillwater, OK, United States

**Keywords:** *Coxiella burnetii*, innate immunity, Toll-like receptors, cytokine—immunological terms, inflammasome, autophagia, immunotherapeutics, experimental model

## Abstract

*Coxiella burnetii* is an obligate intracellular Gram-negative bacterium and the causative agent of a worldwide zoonosis known as Q fever. The pathogen invades monocytes and macrophages, replicating within acidic phagolysosomes and evading host defenses through different immune evasion strategies that are mainly associated with the structure of its lipopolysaccharide. The main transmission routes are aerosols and ingestion of fomites from infected animals. The innate immune system provides the first host defense against the microorganism, and it is crucial to direct the infection towards a self-limiting respiratory disease or the chronic form. This review reports the advances in understanding the mechanisms of innate immunity acting during *C. burnetii* infection and the strategies that pathogen put in place to infect the host cells and to modify the expression of specific host cell genes in order to subvert cellular processes. The mechanisms through which different cell types with different genetic backgrounds are differently susceptible to *C. burnetii* intracellular growth are discussed. The subsets of cytokines induced following *C. burnetii* infection as well as the pathogen influence on an inflammasome-mediated response are also described. Finally, we discuss the use of animal experimental systems for studying the innate immune response against *C. burnetii* and discovering novel methods for prevention and treatment of disease in humans and livestock.

## Introduction

*Coxiella burnetii* (order Legionellales) is an obligate intracellular Gram-negative bacterium (Dragan and Voth, 2020) that can be transmitted by aerosol with a very low infectious dose, as 1–10 viable organisms are sufficient to induce in humans an infection *via* the aerogenic route (Moos and Hackstadt, 1987; Sawyer et al., 1987; Gürtler et al., 2014). These characteristics, combined with the stability in the environment, make *C. burnetii* a potential biological weapon (Oyston and Davies, 2011). *C. burnetii* can infect many species in addition to humans, and cattle, goats, and sheep are the main reservoirs (Van den Brom et al., 2015). In animals, infection by *C. burnetii* can be asymptomatic or it can cause problems on the reproductive sphere. Other manifestations include pneumonia and eye infections (Van den Brom et al., 2015). Humans can acquire the infection by inhalation of infected aerosol or after exposure to urine, feces, placenta, sperm, and vaginal secretions of infected animals. In addition, the disease can be developed after consuming infected raw milk (Dabaja et al., 2020), even if the link between infection and clinical disease in humans through consumption of unpasteurised milk and milk products is unclear (EFSA, 2010). Indeed, although milk may contain large amounts of *C. burnetii*, it is probably a minor route of Q fever acquisition (Maurin and Raoult, 1999; Gale et al., 2015).

*C. burnetii* in humans causes Q fever, usually an acute illness (Waag, 2007; Marrie et al., 2015), but in some cases, it can become chronic (Tobin et al., 1982; Oyston and Davies, 2011). *Coxiella burnetii* life cycle comprehends the stages of Small Cell Variant (SCV) and Large Cell Variant (LCV). The first is the infectious stage, found in the environment and resistant to environmental stresses; it is characterized by the synthesis of molecular determinants of SCV differentiation. SCV development involves responses to protect against oxidative and nutritional stress (Sandoz et al., 2016). Some of these proteins are under the control of the alternative sigma factor RpoS, an essential regulator of stress responses and stationary-phase physiology in several bacterial species (Moormeier et al., 2019). These proteins are involved in regulating the remodeling of the peptidoglycan, in facilitating penetration of the cell envelope and promoting both intracellular and extracellular survival (Moormeier et al., 2019). Among them, *cbu0419* gene encodes a peptidoglycan polysaccharide deacetylase (Caufrier et al., 2003), which renders the peptidoglycan resistant to lysozyme, reducing the release of cell wall fragments and thus the probability of detection by cytosolic immune cell sensors (Vollmer and Tomasz, 2000; Boneca et al., 2007; Davis and Weiser, 2011; Kaoukab-Raji et al., 2012). Another factor that is SCV-associated is enhC (*cbu1136*), whose function in *C. burnetii* is unknown, while in *Legionella pneumophila*, it is thought to aid intracellular replication by controlling lytic murein transglycosylase activity and reducing the subsequent release of peptidoglycan peptide fragments into the cytosol that might be recognized by the cytosolic innate immune system (Liu et al., 2012; Moreira and Zamboni, 2012). Another enzyme that is SCV-associated is the deacetylates *N*-acetylglucosamine of peptidoglycan, which in *C. burnetii* also encodes a lytic murein transglycosylase (CBU0925) that is also known to aid penetration of the cell envelope by large and complex structures (Scheurwater and Burrows, 2011).

**Figure 1:**
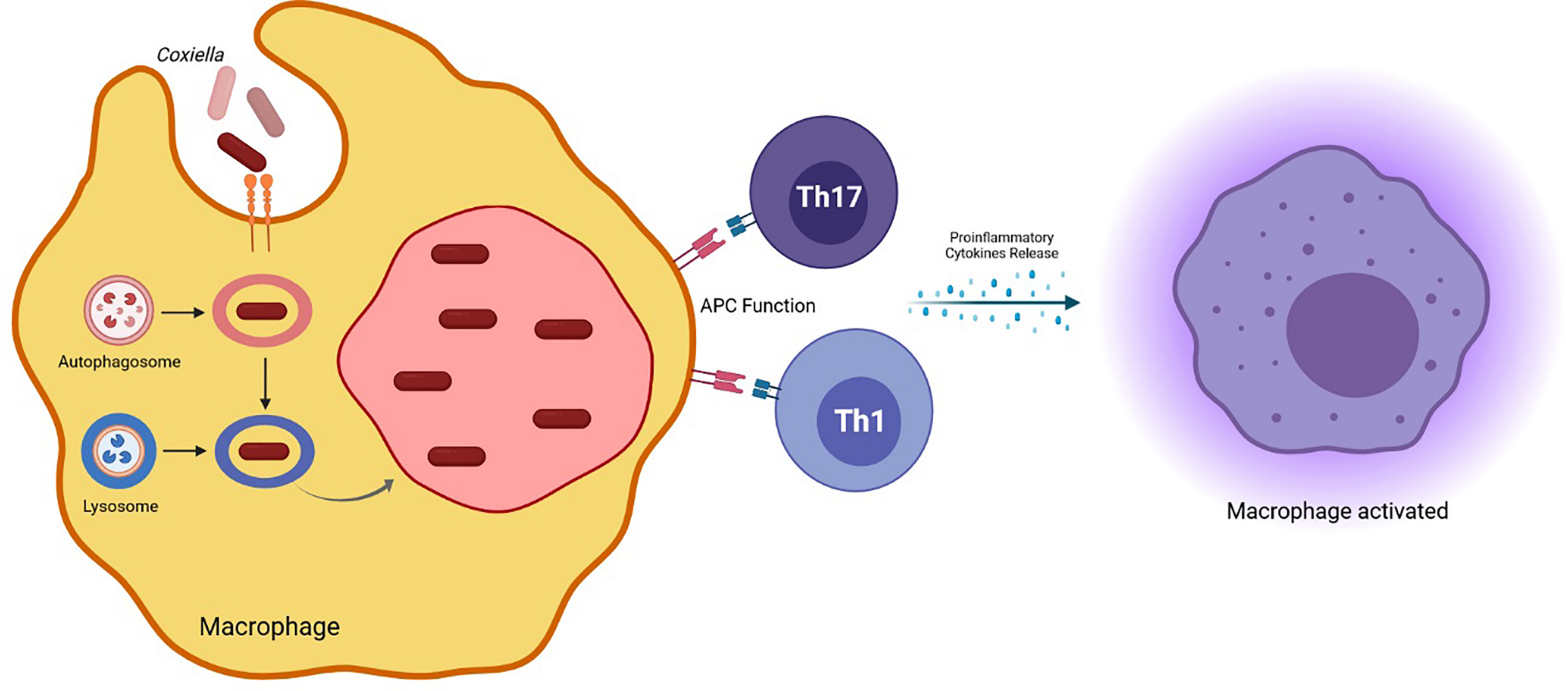
*Coxiella burnetii* phagolysosome formation in the host macrophage and induced effector mechanisms involved in protection against the pathogen.

LCV is the metabolically active form, replicating efficiently in eukaryotic cells, distinguishable by the production of proteins involved in cell division and in intracellular survival (Marrie et al., 2015; Ammerdorffer et al., 2017). SCVs are ingested by the host macrophages and the vacuole containing the bacterium merges with lysosomes to form the phagolysosome (**Figure 1**). Here, SCV becomes LCV, which, in turn, convert back to SCVs (Coleman et al., 2004). During the infection, *C. burnetii* creates a favorable environment inside the cell host to escape from the immune response and to replicate successfully. In particular, *C. burnetii* presents a type IV secretion system (T4SS) essential for renovation of a lysosome into a mature *Coxiella*-containing vacuole (CCV) permissive of intracellular replication. This secretion system has both sequence homology and functional similarity to the defect in organelle trafficking/intracellular multiplication (Dot/Icm) apparatus of *L. pneumophila*, which is designated as the type IVB (T4BSS) and it is involved in creating a vacuole that evades rapid endocytic maturation and matures into an endoplasmic reticulum-derived organelle that supports bacterial replication (Roy and Tilney, 2002).

*Coxiella burnetii* Dot/Icm T4SS consists primarily of bacterial membrane proteins that are assembled into the apparatus used to deliver effector proteins into the cytoplasm of the host cell. These effectors mediate the survival of the host cell and the development of CCV replicative compartment (Lührmann et al., 2017).

The comprehension of the immune response elicited in the host by the pathogen is crucial to understand pathogen transmission, establishment and pathogenesis and for identifying novel checkpoints for pathogen control (Torina et al., 2020a). Innate immunity, in particular, causes a rapid and intense protection for the acute phase of infectious diseases. However, even if effector mechanisms of innate immune responses, displayed by host in short time, are able to inhibit symptoms in the acute infection phases, they do not usually contain the infection (Torina et al., 2020b).

This review is focused on recent advances in the comprehension of innate immune mechanisms involved in protection against *C. burnetii* infection and the strategies elicited by the pathogen to invade the host cells and actively regulate the expression of specific host cell genes to subvert cellular processes. This information could be translated into new intervention for preventing, controlling, and improving Q fever symptoms.

## *Coxiella burnetii* Lipopolysaccharide

One of the major virulence factors of *C. burnetii* is the lipopolysaccharide (LPS). Functional interactions of LPSs and proteins have been thoroughly investigated by glycomics and proteomics studies and a role of this complex emerged not only in growth and development of the microorganism, but also in pathogenesis and immunity of Q fever (Seshadri et al., 2003; Thompson et al., 2003; Skultety et al., 2005; Samoilis et al., 2007; Toman et al., 2009). *C. burnetii* LPS is involved in immune evasion strategies since its peculiar structure leads to repressive effects on the defensive mechanisms of cells (Abnave et al., 2017).

Wild-type *C. burnetii* strains possess a complex full-length LPS with typical O-chain saccharidic units that makes the bacterium virulent (it is the so-called phase I) (Hackstadt, 1986). Following repeated passage in chick embryo yolk sacs or in cell lines, i.e., in the absence of immune system intervention, *C. burnetii* wild-type strains convert to phase II and produce a phase II LPS determining the loss of virulence (Lukácová et al., 2008).

Phase II LPS is easily eliminated by immune response in immunocompetent hosts (Hackstadt, 1990). This passage is accompanied by modifications in both composition and structure of the LPS macromolecule (Toman et al., 2009). LPS II shows a rough (R) structure, unlike LPS I, characterized by a smooth (S) aspect. Moreover, LPS II is characterized by a peculiar composition, which also includes the sugars virenose and dihydrohydroxystreptose, not present in other LPS and which are therefore representative of the *C. burnetii* LPS (Narasaki and Toman, 2012). Phase variation is also used in the diagnosis of Q fever since during acute Q fever, *C. burnetii* induces antibodies against phase II (protein antigens), while chronic Q fever, often manifested as endocarditis, is associated with the production of high titers of antibodies directed against phase I (LPS antigen) (Peacock et al., 1983).

Truncated LPS is responsible for the avirulence of the Nine Mile phase II strain. Moreover, since phase I LPS of *C. burnetii* possesses low antigenic and immunogenic properties (Hitchcock et al., 1986), this LPS–protein complex is also used as a vaccine against Q fever.

Though LPS I and LPS II are weak endotoxins, their ability to induce TNF-α has been reported (Toman et al., 2009). In TNF-α and many other cytokines (IL1b, IL10, etc.), overproduction is involved (Dellacasagrande et al., 2000a) in *C. burnetii* survival inside the patient monocytes and may be related to specific inflammatory syndrome of Q fever endocarditis consisting of an increase in circulating TNF-α without variations in cytokine antagonist (Capo et al., 1999a). TNF production did not directly reflect the virulence of *C. burnetii* strains since avirulent organisms induced TNF production. The overproduction of TNF stimulated by avirulent *C. burnetii* is probably related to the increase in bacterial binding to monocytes and not to the potency of their LPS (Dellacasagrande et al., 2000b), even considering that the interaction of avirulent variants with monocytes was dramatically more efficient than that of virulent organisms (Capo et al., 1999b).

## *Coxiella burnetii* Infection and Host Cell Toll-Like Receptors

Most pathogens are initially recognized by the innate immune system through one or more Toll-like receptors (TLRs) (Medzhitov, 2007), a family of pattern recognition receptors present on macrophages and other cells acting in innate immunity. TLRs are able to recognize different microbial structures/patterns, thus intervening in the early immune responses to several etiologic agents (Ramstead et al., 2016). Their activity is often mediated by MyD88, which acts as an adapter involved in signal transduction (Janssens and Beyaert, 2002).

Different studies investigated the role of TLR4 and TLR2 in *C. burnetii* infections (Honstettre et al., 2004; Zamboni et al., 2004; Meghari et al., 2005; Shannon et al., 2005). It was reported that phase I *C. burnetii* lacks the ability to stimulate TLR2 and TLR4 as a possible immune evasion strategy (Shannon et al., 2005).

Moreover, it was reported that *C. burnetii* LPS is able to avoid the activation of p38α-MAPK through the TLR4 recruitment. In particular, the re-organization of the macrophage cytoskeleton by *C. burnetii* LPS disrupts the interaction between TLR-2 and TLR-4, which is necessary for TLRs signaling in case of recognition of pathogenic *C. burnetii* LPS. Blocking the actin-mediated cytoskeleton re-organization was able to restore *C. burnetii*-induced TLR-2/TLR-4 association and the activation of p38α-MAPK (Conti et al., 2015).

A study investigating the involvement of TLR2, TLR4, and MyD88 in pulmonary *C. burnetii* infection found a different role for these factors according to the site of infection. Indeed, TLR2 and TLR4 were not necessary to reduce the growth of the pathogen following peripheral infection and to elicit the inflammatory response in the lungs following pulmonary infection. However, they were able to limit bacterium replication in the lung and spleen when pulmonary infection occurred. Moreover, MyD88 was involved in the infection-related morbidity (Ramstead et al., 2016).

Other factors involved in the innate immune response against microorganisms are nucleotide-binding oligomerization domain receptor 1 (NOD1), NOD2, and the mitogen-activated protein kinases. Inhibition of TLR2 and mitogen-activated protein kinases as well as polymorphisms in TLR1 and NOD2 decreased cytokine responses in *C. burnetii*–stimulated human peripheral blood mononuclear cells (PBMCs), confirming the important role of both TLR1/TLR2 heterodimer and NOD2 for cytokine response induction against *C. burnetii* in humans (Ammerdorffer et al., 2015). In a subsequent study, the same authors found that the absence of TLR10 resulted in increased cytokine responses following *C. burnetii* infection. Similarly, the occurrence of SNPs in TLR10 in healthy volunteers increased cytokine production upon *C. burnetii* stimulation, suggesting an inhibitory effect of TLR10 (Ammerdorffer et al., 2016).

A different response of human TLR4 (hTLR4) and mouse TLR4 against differently hypo-acylated LPS has been reported. Since it is known that *C. burnetii* LPS is hypo-acylated and heavily glycosylated, a recent study investigated susceptibility to *C. burnetii* infection in mice expressing hTLR4. Although mice expressing hTLR4 and the human myeloid differentiation factor 2 (MD-2) adaptor protein showed a similar response with respect to wild-type mice, the authors observed differences in bacterial burdens in tissues and an increase in lung pathology in the first ones. An alteration of cytokine response was observed in hTLR4/MD2 mice, which showed the production of cytokines involved in myeloid cell recruitment accompanied by neutrophil recruitment to the lung. These responses were related to the subsequent observed increased bacterial load and worst pathology in the lung (Robison et al., 2019).

## Macrophage Role in *C. burnetii* Infection

*Coxiella burnetii* is able to apply several survival strategies to persist within myeloid cells by subverting their microbicidal activity (Ghigo et al., 2009). An interesting study reported the innate immune reactions of neutrophils and macrophages within the lungs elicited following an aerosol-mediated *C. burnetii* infection. These authors reported that both virulent *C. burnetii* Nine Mile phase I (NMI) and avirulent Nine Mile phase II (NMII) were able to infect neutrophils, but the infection was limited since a low infection rate was observed. In addition, a possible *C. burnetii* evasive strategy to infect macrophage emerged since neutrophils did not kill *C. burnetii* and the bacterium inside neutrophils was still able to infect and replicate within macrophages. An early neutrophil-mediated immune response against aerosolized *C. burnetii* was detected as SCID mice exposed to aerosolized *C. burnetii* showed a delay in the influx of neutrophils into the lungs, both in case of NMI and in case of NMII infection (Elliott et al., 2013).

Human alveolar macrophages (hAMs) have been used as a model for defining novel innate immune responses to *C. burnetii*. These cells allowed the replication of *C. burnetii* in large, typical CCV, and in non-fused, atypical vacuole harboring replicating *C. burnetii* and recruiting lipid droplet. A functional type IV secretion system was necessary for the CCV formation and bacterial growth, suggesting production of effector proteins by *C. burnetii* able to control macrophage functions. Avirulent *C. burnetii* promoted the activation of pro-survival kinases, a short-term phosphorylation of stress-related factors and a robust, early pro-inflammatory response through the secretion of TNF-α and IL-6. On the contrary, virulent isolates elicited reduced secretion of these cytokines. Finally, IFN-γ treatment of infected hAMs was able to control the intracellular replication of *C. burnetii* (Graham et al., 2013).

*Coxiella burnetii* showed a limited ability of alveolar macrophage activation, as it was also supported by studies showing that the interactions between *C. burnetii* and the C-type lectin surfactant protein D (SP-D), a member of the innate immune response in different mucosal surfaces and in the lungs, result in bacterial aggregation provoking pathogen killing and clearance. It was observed that SP-D is able to bind *C. burnetii* in a calcium-dependent manner, but no bacterial aggregation or bactericidal effects have been observed. SP-D interactions with *C. burnetii* decreased the infectivity of *C. burnetii* towards mouse alveolar macrophage cells, probably due to a significant reduction in its adherence and phagocytosis. However, SP-D does not alter classical activation of alveolar macrophages (Soltysiak et al., 2015).

*Coxiella burnetii* phase II infection of primary murine alveolar macrophages (AMs) has been investigated *in vitro* (Fernandes et al., 2016), resulting in a pronounced M2 polarization of AMs and in the establishing of a highly permissive condition to *C. burnetii* multiplication *in vitro*. Murine AMs showed an increased susceptibility to infection in comparison to primary bone marrow-derived macrophages. Moreover, AMs from Nos2 or IFNγ knockout (KO) mice were significantly more permissive than wild-type cells when cultured in the absence of IFNγ and nitric oxide synthase 2 (NOS2). In contrast, AMs from Il4^-^/^-^ mice (in C57BL/6 mouse genetic background) were more restrictive to *C. burnetii* replication. These results showed that M2 polarization is relevant to elicit the permissiveness of AMs to *C. burnetii* replication (Fernandes et al., 2016).

It was reported that *C. burnetii* NMII strain was able to replicate in both primary macrophages from C57BL/6J mice obtained differentiating bone marrow cells with recombinant murine macrophage-colony stimulating factor (M-CSF) as well as in C57BL/6J myeloid progenitors immortalized with an estrogen-regulated *Hoxb8* oncogene. These studies indicated that both these *ex vivo* models can be useful to investigate the interplay between *Coxiella* and macrophages (Cockrell et al., 2017).

*Coxiella burnetii* infection was also analyzed in CD14^+^ macrophages collected from full-term placentas. The bacterium was detected in these macrophages after 4 h culture and was eliminated after 9 days. *C. burnetii* infection did not prevent placental macrophages from generating multinucleated giant cells, and it was able to induce an inflammatory profile, in particular stimulating IFN-γ production. Indeed, *C. burnetii* clearance was clearly correlated to IFN-γ production (Mezouar et al., 2019a).

An involvement of mast cells (MCs) has been supposed in Q fever pathophysiology since a decrease in MC progenitor count was observed in Q fever patients together with an increase of serum tryptase levels, which are markers of MCs activation (Mezouar et al., 2019b).

## Cytokines in Q Fever

Cytokines are proteins involved in immunity and inflammation, which are mainly produced by monocytes and lymphocytes in response to an immune insult. Since *C. burnetii* is an obligate intracellular pathogen, it causes alterations in host immune response to survive inside this cell, and the cytokine response is significantly affected by modifications induced by the bacterium (Amara et al., 2012). For example, PBMCs from patients affected by Q fever Fatigue Syndrome (QFS), a debilitating fatigue syndrome lasting 5 to 10 years or longer after the acute illness, showed increased IL-6 and IFN-gamma and reduced IL-2 levels compared to controls (Penttila et al., 1998).

The role of cytokines in the pathogenesis of the acute and chronic Q fever and in the development of the QFS has been investigated. For instance, it has been hypothesized that cytokines may be responsible of persistent fever in patients affected by acute Q fever, which scarcely respond to antibiotic therapy (Lai et al., 2010). Moreover, serum levels of specific cytokines have been suggested as markers of acute or chronic Q fever.

It has been proposed that acute *C. burnetii* infection may cause an epigenetic remodeling of the promoter regions of pro-inflammatory cytokine genes, which determines an impaired inflammatory response. In particular, QFS patients affected by frequent and severe infections in the upper respiratory tract show a reduced cytokine response, probably due to a long-term change in monocytes induced by *C. burnetii* (Raijmakers et al., 2019).

*Coxiella burnetii* isolated from cattle induced higher levels of IL-1β, TNF-α, and IL-22 than bacteria isolated from goats, sheep, and chronic Q fever patients, which probably explains the low incidence of human outbreak associated with bovine disease. Cytokine responses depended on host origin more than on *C. burnetii* genotypes (Ammerdorffer et al., 2017).

Moreover, viable *C. burnetii* induces lower levels of TNF-α, IL-1β, IL-6, and IL-10 but similar levels of IFN-γ, IL-17, IL-22, and CXCL9 (IFN-γ-dependent chemokine) compared to the heat-killed bacteria. In contrast, the adaptive response is similar in both cases (Jansen et al., 2018).

In Q fever, IFN-γ promotes *C. burnetii* killing by the induction of TNF-mediated apoptosis in infected monocytes (Dellacasagrande et al., 1999; Dellacasagrande et al., 2002). In particular, IFN-γ is significantly increased in acute but not in vascular chronic Q fever patients compared to people with past infection, whereas IL-18 shows a similar rise in both acute and chronic patients. The IFN-γ-inducible protein 10 (IP-10) and the TGF-β instead are lower in patients affected by chronic vascular infection, suggesting that chronic patients had an impaired Type I immune response against *C. burnetii* (Pennings et al., 2015).

Patients affected by chronic Q fever showed intact IFN-γ response, so defects concerning this pathway are not involved in progression to chronic infection. Instead, since polymorphisms in the IL-12p40 gene are associated with chronic Q fever, the IL-12/IFN-γ pathway may play a role in this progression (Schoffelen et al., 2017). The QFS patients show similar levels of IFN-γ and decreased IFN-γ/IL-2 ratio compared to those affected by chronic Q fever. Symptom duration is positively correlated with IL-2 production and negatively correlated with the IFN-γ/IL-2 ratio, suggesting an impaired cell-mediated response in QFS (Keijmel et al., 2016).

Type I IFNs (IFN-α, β, κ, ω, τ, and ϵ) and other interferons are important in immune response against *C. burnetii* (Snyder et al., 2017). IFN-α administration at peritoneal level exacerbated disease, while at the lung level (site of infection), it reduced bacterial replication (Hedges et al., 2016). The negative impact of the peripheral delivery may be explained by the fact that type I IFNs reduce the inflammatory cytokine expression, whereas the beneficial effect of lung administration suggests that aerosolized type I IFNs may be an effective alternative to antibiotics to treat affected patients. There was also a report of an interaction between *C. burnetii* and plasmacytoid dendritic cells (pDCs), which play a key role in antiviral immunity *via* the production of I IFNs. *C. burnetii* was able to stimulate pDCs increasing the expression of activation and migratory markers and upregulating genes encoding for pro-inflammatory cytokines, chemokines, and type I IFN. Type I IFN upregulation was related to the increase in IFN-α release by *C. burnetii*-stimulated pDCs (Ka et al., 2016).

*Coxiella burnetii* also stimulates IL-10 production, a cytokine crucial for bacterial replication inside of monocytes. The mechanism involved probably consists in monocyte deactivation mediated by TNF suppression. It has been suggested that IL-10 overproduction promotes progression to chronic Q fever (Ghigo et al., 2001). Monocytes from patients affected by Q fever endocarditis instead show increased TNF production, which is associated with impaired *C. burnetii* elimination (Dellacasagrande et al., 2000b). Other studies, conversely, reported that the production of TNF is required to restrict *C. burnetii* Nine Mile II replication within macrophages (Bradley et al., 2016).

Q fever patients with hepatitis show serum cytokines levels higher than those affected by pneumonia or fever, and TNF and IL-10 are increased in patients with valvulopathy and extremely high in case of endocarditis. These observations indicate that acute Q fever is associated with cytokine overproduction and that TNF may be a marker of chronic evolution of Q fever (Honstettre et al., 2003). Besides, the activation marker of B cells sCD23 in Q fever endocarditis patients is significantly higher than in acute patients and controls, suggesting that it may be a marker of the Q fever endocarditis (Capo et al., 1999a).

Serum levels of cytokines such as IFN-γ and IL-6 may be helpful for distinguishing between past and chronic disease as well as for diagnosis of acute Q fever. For example, it has been observed that an IFN-γ/IL-2 ratio > 11 is strongly suggestive for chronic Q fever (Schoffelen et al., 2014b). Likewise, in sheep, goats and she-camels, cytokines, acute phase proteins, and oxidative stress markers may be useful tools to diagnose the Q fever, since animals that aborted due to coxiellosis show a significant increase of the proinflammatory cytokines (El-Deeb et al., 2019).

Finally, IFN-γ and IL-10 serum levels in dairy cattle may be helpful to value the health status of seropositive animals and to monitor response to vaccine (Małaczewska et al., 2018).

## Apoptosis and Inflammasome in *Coxiella burnetii* Infections

Apoptosis is a programmed cell death process essential for the immune system homeostasis, acting also as a scavenger for damaged or infected cells (Byrne and Ojcius, 2004). Apoptosis can be triggered through two different pathways called the extrinsic and intrinsic pathway. In the first pathway, several ligands are involved, such as Fas Ligand (FasL) and TNF-related apoptosis-inducing ligand (TRAIL), their death receptors, and some adaptor proteins, which activate caspases (cysteinyl aspartate proteases). In the second pathway, the activation of the Bax group of proteins plays a crucial role. Their oligomerization leads to the permeabilization of the mitochondrial membrane, the resulting cytochrome *c* release, and the activation of effector caspases (caspases 3, 6, and 7). In detail, caspase 3 induces robust proteolysis and chromatin fragmentation (Enari et al., 1998; Cory and Adams, 2002).

Nowadays, it is well known that some pathogens are able to interfere positively or negatively with the apoptotic process through different pathways, such as *Chlamydia pneumoniae*, *Rickettsia rickettsii*, and *Toxoplasma gondii.* They act, for example, by degrading pro-apoptotic proteins, inducing the transcription of some genes encoding anti-apoptotic proteins or inhibiting the activation of the intrinsic pathway (Clifton et al., 1998; Byrne and Ojcius, 2004; Fischer et al., 2004; Carmen et al., 2006).

*C. burnetii*, as other intracellular pathogens, need to hinder the apoptosis process to survive and to establish a favorable environment inside the host cell. It has been seen that cells infected by *C. burnetii* show different susceptibility to apoptosis depending on their type, stage of maturation, and surface receptors expressed (Schoenlaub et al., 2016). For example, infection by NM II *C. burnetii* in murine bone marrow neutrophils inhibits apoptosis (Cherla et al., 2018), whereas in peritoneal B1a cells from the same species, it induces cell death (Schoenlaub et al., 2016). Similarly, Zhang et al. (2012) obseved that in human monocytic THP-1 cells, early *C. burnetii* infection induces apoptosis by a caspase-independent pathway involving translocation of Bcl-2-associated X protein (BAX) and Apoptosis-inducing factor (AIF), respectively, to the mitochondria and to the nucleus. The authors suggested that mantaining a certain level of apoptosis may be useful for *C. burnetii* to establish persistent infection.

It has been seen that *C. burnetii* infection affects both intrinsic and extrinsic apoptosis pathway (Bisle et al., 2016; Cordsmeier et al., 2019). Some experimental evidences showed that *C. burnetii* inhibits the activation of the host cell apoptotic process preventing the release of cytochrome *c* and the subsequent activation of caspase 3/7, probably through the upregulation of *A1/bfl-1* and *c-IAP2* (Lührmann and Roy, 2007; Voth et al., 2007). Effectors needed to inhibit apoptosis are injected inside the host cell through the type IVB secretion system (T4BSS) (Lührmann et al., 2010).

However, it is well known that microbial activation of TLRs in macrophages commonly leads to the induction of anti-apoptotic gene expression, suggesting that the *C. burnetii* could interfere with host cell survival through the activity of some specific bacterial protein produced upon the infection.

Among the *C. burnetii* proteins involved in the anti-apoptotic process, AnkG, CaeA, and CaeB are the most studied. AnkG is a 388-amino acid protein that belongs to the Ankyrin repeat family and is capable of blocking the p32-mediated apoptosis pathway in mammalian cells (Lührmann et al., 2010). It has been seen that the amino terminal 1–69 region codify a domain that is both necessary and sufficient to interact with p32 and to inhibit the apoptosis, whereas truncated variants are not able to block apoptosis. Moreover, to prevent pathogen-induced cell death, AnkG needs to be localized in the nucleus (Eckart et al., 2014; Schäfer et al., 2017; Schäfer et al., 2020).

CaeB is a T4BSS effector protein, which, in stressed mammalian cells, targets the endoplasmatic reticulum stress sensor IRE1, thereby inhibiting the ER stress-induced apoptosis. Due to its activity, CaeB is involved in pathogenity *in vivo* (Friedrich et al., 2021).

In contrast to CaeB, CaeA affects both intrinsic and extrinsic apoptosis acting respectively on caspases and Fas pathways. An EK (glutamic acid/lysine) repetition motif is believed to be essential for CaeA anti-apoptotic activity (Bisle et al., 2016; Raghavan, 2016).

Finally, apart from those mentioned above, another *C. burnetii* effector worth mentioning is IcaA, secreted, similarly to AnkG, CaeA, and CaeB, by the Dot/Icm type IV secretion system. This protein is responsible for caspase-11 inhibition and plays an important role in preventing pyroptosis, which is an inflammatory cell death pathway massively involved in infection control (Cunha & Zamboni, 2013; Cunha et al., 2015; Cordsmeier et al., 2019).

In addition, *C. burnetii*, as well as other bacteria, implements several mechanisms to evade inflammasome activation and pyroptosis induction (Cunha and Zamboni, 2013).

Inflammasome is a protein scaffold involved in modulation of caspase-1 and in the maturation of IL-1β and IL-18 (Torina et al., 2020b). It is activated during infections by several pathogens, including Gram-negative and Gram-positive bacteria, virus, fungi, and protozoa (Hayward et al., 2018; Scorpio et al., 2018). It is generally activated by different Pathogen-Associated Molecular Patterns (PAMPs) or Damage-Associated Molecular Patterns (DAMPs) through various Nod-like receptors (NLRs), such as NLRP3. Inflammasome activation induces several processes, including the release of Caspase-1 active form. The protease allows pro-IL1β/pro-IL18 maturation and the release of active IL1β and IL18 with pyroptotic, or inflammatory, cell death induction.

*Coxiella burnetii*, as well as other bacteria, implements several mechanisms to evade inflammasome activation and pyroptosis induction (Cunha and Zamboni, 2013). For example, a study found that *C. burnetii* was able to prevent the non-canonical activation of the NLRP3 inflammasome mediated by caspase-11 in case of successive infection with other bacteria such as *Escherichia coli* or *L. pneumophila*. This activity was related to a novel *C. burnetii* gene (*IcaA*) codifying a protein interfering with caspase activation and with the non-canonical activation of the inflammasome mediated by caspase-11 (Cunha et al., 2015).

*Coxiella burnetii* is not involved in caspase-1 activation, IL-1b secretion, or cell death during the *in vitro* infection of murine macrophages. Moreover, *C. burnetii*-infected cells have no evident NLRP3 or ASC (apoptosis-associated speck-like protein containing a carboxy-terminal caspase activation and recruitment domain [CARD]) foci, indicating its ability to avoid cytosolic detection. *C. burnetii* is not able to inhibit and instead potentiates Caspase-1-mediated cell death, upregulating pro-IL-1b and priming NLRP3 inflammasomes *via* TLR2 and MyD88 signaling (Delaney et al., 2021).

## Autophagy

*Coxiella burnetii* can subvert the autophagy process to obtain a better bacterial replication activity taking advantage of the host’s cellular response (Castrejón-Jiménez et al., 2015). It was argued that the autophagic compartments rich in nutrients (cell degradation products, small peptides, amino acids, etc.) such as those of the host cell autophagosomes, contribute to the conversion of SCV to LCV. Therefore, the morphogenesis and multiplication of the bacterium depend not only on the pH of the vacuoles, but also on the accessibility of nutrients (Gutierrez and Colombo, 2005; Colombo et al., 2006).

The role of the bacterial Dot/Icm type IV secretion system, through which *C. burnetii* translocates effector substrates from the bacterial cytosol directly into the cytosol of the host eukaryotic cell, has been investigated. Translocated effectors are involved in the pathogenesis mechanisms and therefore in the progression of infection. Through these effectors, *C. burnetii* modulates several cellular activities in order to maintain the infection. These activities include apoptosis suppression, lipid metabolism, and membrane trafficking alteration, gene transcription modification through the interaction of these effectors directly with targets in the nucleus, etc. (van Schaik et al., 2013).

One of these proteins is Cig2, also known as vacuolar protein B (CvpB) (Martinez et al., 2016). This *C. bunetii* effector interacts with phosphoinositides on host cell membranes and manipulates phosphatidylinositol 3-phosphate metabolism for optimal *Coxiella*-containing vacuole (CCV) development, promoting the fusion between autolysosomes and CCV and maintaining it in an autolysosomal stage. This allows distinguishing the CCV from the other vacuoles containing other pathogenic bacteria, which become targets of the host’s defensive pathway. This is a mechanism through which *C. bunetii* is able to subvert the host phagocytosis pathway in its favor, influencing the host’s immune response (Kohler and Roy, 2015).

Another study reported that GTPases of the Rho family have an important role in *C. burnetii* phagocytosis. More specifically, the active forms of the members of the Rho family RhoA, Cdc42, and Rac1 are involved in the entry of *C. burnetii* into the host cell, regulating the rearrangement of actin, necessary for the internalization process. The authors also showed that the effectors of RhoA mDia1 and ROCK are involved in the signal transduction mechanism, which favors the entry of *C. burnetii* (Salinas et al., 2015).

A recent study identified host proteins required for Dot/Icm effector translocation and for endocytic trafficking of the *C. burnetii*-containing vacuole to the lysosome. Moreover, many of the lysosomal proteins contribute to the virulence of *C. burnetii*, including the lysosomal serine protease TPP1. This tripeptidyl peptidase 1, also known as CLN2, is selectively transported to the lysosome by a lysosomal receptor, where it generates tripeptides from degraded proteins within the lysosome (Qian et al., 2008). The authors demonstrated that *C. burnetii* is capable of detecting specific amino acids present in lysosomes and of upregulating gene expression required for the activity of the Dot/Icm system itself (Newton et al., 2020).

A role also for galectin proteins, β-galactoside-binding lectins involved in many biological processes (Blanda et al., 2020), has been reported. Galectin-3, -8, and -9 monitor bacteria vacuolar rupture, and endosomal and lysosomal loss of membrane integrity through binding of host glycans exposed in the cytoplasm after membrane damage (Mansilla Pareja et al., 2017). Moreover, vacuoles containing *C. burnetii* are characterized by a reversible recruitment of members of the endosomal sorting complex required for transport (ESCRT) and the interference with this recruitment reduces intravacuolar bacterial replication (Radulovic et al., 2018).

## Innate Immunity in Chronic Q Fever

Patients affected by Q fever usually recover entirely through antimicrobial therapy or even without any treatment, but in less than 5% of cases they develop chronic infection, a serious disease difficult to treat (a minimum of 18 months of antimicrobial therapy is required). Endocarditis and vascular infection are the most frequent clinical manifestations that can be lethal if untreated (Mazokopakis et al., 2010). Moreover, patients returning from *C. burnetii* acute infection can develop a chronic fatigue syndrome (Wildman et al., 2002; Morroy et al., 2016).

Mechanisms involved in the establishment of chronic infection are unknown, but it has been suggested that the chronicity may be due to a specific immune deficiency (e.g., lack of IFN-γ) and not to a general immunodeficiency. Pregnant women, people affected by heart valve disorders, and cancer patients are most at risk to develop chronic infection.

Several studies focused on the role of the immune response, and defects in both innate and adaptive immunity have been linked to the risk of developing the disease (Capo and Mege, 2012).

For example, it was reported that monocytes from patients with chronic Q fever in evolution, who do not control the infection, exhibited defective phagosome maturation and impaired *C. burnetii* killing. Both responses were restored in patients recovering from Q fever. A significant correlation was reported between phagosome maturation and *C. burnetii* killing. Defective phagosome maturation and impaired *C. burnetii* killing were induced by interleukin (IL)-10 addition to monocytes from convalescent patients and were restored by IL-10 neutralization in chronic Q fever in evolution (Ghigo et al., 2004).

The defective granuloma formation, an ineffective high level of TNF, a lack of antimicrobial activity of monocytes, and the failure of T-cell response are other examples of anomalies that appear involved in the chronic infection establishment. Genetically modified mice overexpressing IL-10 have been used as a model to analyze the role of IL-10 in chronic Q fever development. It has been demonstrated that IL-10 overproduction suppresses the microbicidal pathway (NO synthase and inflammatory cytokines), making the macrophages incapable of killing *C. burnetii*. This altered immune response causes sustained *C. burnetii* burden in tissues, high levels of antibody, and impaired granuloma formation, which are typical of the chronic Q fever (Meghari et al., 2008).

Some studies (Marmion et al., 2009; Sukocheva et al., 2010) performed by inoculating SCID mice with samples (bone marrow, PBMC, or heart valve) coming from Q fever patients showed the persistence of the so-called “Immunomodulatory complex” (IMC) even after a long time away from acute infection. This is composed by non-infective and non-biodegraded *Coxiella* cell components, including *C. burnetii* antigens, specific LPS, and traces of genomic DNA. The persistence of the IMC involve the capacity to pass repeatedly into the macrophages, and it induces an abnormal immune response, which may explain the development of chronic sequelae as the long fatigue syndrome after the acute infection in some patients.

Th-1 and IFN-γ response is another aspect that has become crucial to control acute *C. burnetii* infection. The development of the chronic Q fever reflects indeed its failure, whereas the antibody response seems less important (Eldin et al., 2017). The IFN-γ inducible chemokine CXCL9 is higher in sera from chronic Q fever patients than in people healed from *C. burnetii* infection, and so it has been proposed as a chronic Q fever biomarker (Jansen et al., 2017a). Even genetics host factors have a role in chronic Q fever progression. For example, SNPs in genes crucial for phagolysosome maturation, bacterial killing, and autophagy are involved in higher or decreased susceptibility to chronic Q fever (Jansen et al., 2019).

A further example can be found in the genes coding for matrix metalloproteinases (MMPs). *Coxiella burnetii* in fact induces their overexpression, contributing to the onset of chronic Q fever. Moreover, SNPs in MMP genes are more common in patients affected by chronic infection than in healthy people, so they can be considered as a risk factor (Jansen et al., 2017b).

Persistent infection by *C. burnetii* is a serious problem also for animal health. Affected Cows, sheep, and goats indeed show reproductive troubles, which result in economic damage for the farmers. In cattle, chronic Q fever often causes ipofertility, endometritis, and low birth weight, whereas goats and sheep show more frequent abortion and premature delivery. In another study (De Biase et al., 2018), the correlation between *C. burnetii* infection and reproductive problems in cows was proven through the detection of the bacterium inside the uterine macrophages of cattle affected by chronic endometritis and low fertility and PCR and aerobic culture negative for other pathogens.

In addition, another important problem relating to affected animals is that they expel *C. burnetii* through milk, feces, vaginal mucus, and birth products, which are vehicles for spreading the infection between the livestock and to the humans. Cattle, goats, and sheep are indeed considered as the most important reservoirs for the human infection (Georgiev et al., 2013; Pouquet et al., 2020).

## New Immunotherapeutic Interventions in Experimental Models of *C. burnetii* Infections

Model organisms and cell culture are relevant for understanding complex biological mechanisms, as the immune response and the interaction between pathogen and host (Metters et al., 2019). Animals belonging to invertebrates, small mammals, and non-human primates have been used to analyze the interaction between *C. burnetii* and host cell, the course of disease, and the role of innate and adaptive immunity against the infection (Bewley, 2013) (**Table 1**).

**Table 1:** Experimental models and results of *C. burnetii* infection.

Experimental model	Purpose	Infection route	Effect	Ref.
*Caenorhabditis elegans*	To investigate the role of the host innate immunity	Feeding	Exposure to live bacterium caused clear pathologic effects (as intestinal distension and anal deformation) on *C. elegans* No problems after exposure to the killed bacterium Some mutants *C. elegans* showed reduced lifespan or slighter infection signs	Battisti et al., 2017
*Drosophila melanogaster*	To investigate the role of the host innate immune response and of the *Coxiella* type 4 secretion system (T4SS) in Q fever pathogenesis	Injection	*D. melanogaster* lacking in Eiger, a tumor necrosis factor (TNF) superfamily homolog in *Drosophila*, less susceptible to the infection and *Coxiella*-infected flies exhibit reduced mortality from infection. *Coxiella* T4SS is critical for the formation of the *Coxiella*-containing vacuole and establishment of infection in *Drosophila*.	Bastos et al., 2017
*Galleria mellonella*	To investigate susceptibility to infection with both *C. burnetii* NMI and NMII. To characterize the role of the type 4 secretion system in *C. burnetii* NMII	Injection	*G. mellonella* susceptible to lethal infection with *C. burnetii* without significant difference in virulence between *C. burnetii* NMI and NMII strains. *C. burnetii* induce dose-dependent killing of *G. mellonella*. Dot/Icm type 4 secretion system (T4SS) is required for *C. burnetii* NMII virulence.	Norville et al., 2014
*Galleria mellonella*	To identify virulence genes of *C. burnetii* NMII	Injection	*Coxiella* strains exhibit various levels of pathogenicity for *G. mellonella.* Tetracycline increased the mean time to death and decreased the lethal effect of *Coxiella* strains.	Selim et al., 2018
*Galleria mellonella*	To study the intracellular growth and identify potential virulence factors. To map the transcriptome of *C. burnetii* during the infection	Injection	*C. burnetii* replicate in *G. mellonella* hemocytes and occupy a vacuole similar to the CCV seen in infected mammalian phagocytes. Identification of genes significantly upregulated or downregulated at different days postinfection.	Kovacs-Simon et al., 2020
*Ixodes scapularis*	To characterize host–pathogen interactions	*In vitro* using IDE8 cells	Development of an *in vitro* model	Herrin et al., 2011
Inbred laboratory mice	To evaluate susceptibility of inbred mice to infection by phase I *C. burnetii*	Inoculated intraperitoneally	Resistant, of intermediate sensitivity, and sensitive inbred mice were identified. Among the susceptible ones, BALB/c mice were infectable and showed signs of disease, A/J mice had the highest mortality rate	Scott et al., 1987
IFN-γ knockout mice, BALB/c, Toll-like receptor 2 knockout mice and C57BL/6	To verify if avirulent *C. burnetii* NM II could elicit disease in immunodeficient mice	Inoculated intraperitoneally	Fever and splenomegaly in immunodeficient mice, minimal symptoms in immunocompetent mice. Immunodeficient mice were protected against secondary infection: possible use of NMII for vaccine development against *C. burnetii*	Ochoa-Repáraz et al., 2007
My-D88-deficient mice	To study early *C. burnetii* infection and prevention of infection spreading	*In vitro* infection of bone marrow-derived macrophages	MyD88-dependent signaling is essential for early control of *C. burnetii* infection and to prevent systemic dissemination.	Kohl et al., 2019
BALB/cJ Mice	To study Q fever in pregnancy.	Inoculated intraperitoneally	Elevated bacterial load in the placenta, associated with still birth, perinatal death and high abortion rate, decreased birth rate	Baumgärtner and Bachmann, 1992
Female BALB/c	To investigate manifestations of perinatal Q fever	Intraperitoneal infection	Persistent infection associated with abortion and perinatal death, increased risk of endocarditis in pregnant mice	Stein et al., 2000
Mice overexpressing IL-10	To clarify the role of host immune response in chronic Q fever	Intraperitoneal and intratracheal routes	Alteration in granuloma formation, elevated antibodies levels, and marked *C. burnetii* load in the tissue, consistent with human chronic infection characteristics	Meghari et al., 2008
C57/BL6 mice	To investigate the role of sex hormones in the pathogenesis of *C. burnetii* infection.	Injected intraperitoneally	*C. burnetii* load and granuloma numbers reduced in females. Ovarectomized mice increased presence of bacterium and granuloma, to a male similar level. Ovarectomized mice treated with 17β- estradiol reduced both bacteria and granuloma numbers	Leone et al., 2004
Guinea pigs	To investigate the pathophysiologic features of Q fever.	Inoculated intraperitoneally	Liver, spleen, heart, and lungs were the most affected organs; granulomas detected in liver and spleen, infiltrations of mononuclear cells detected in lung alveoli and in the heart. No valvular damage found, except for animals with formerly electrocoagulated heart valves	Heggers et al., 1975
Inbred strains of mice, and outbred guinea pigs	To investigate the susceptibility to *C. burnetii* infection	Several routes	Guinea pigs more susceptible with respect to mice, which showed more diffused lesions.	Scott et al., 1978
Nonhuman primates	Study of the pathogenesis of infection with *Coxiella burnetii*	Aerosol	Acute illness as in humans, (hepatitis, pneumonia, and blood alterations as marked AST increase), antibodies against the phase II develop first and achieve higher levels than antibodies against the phase I	Gonder et al., 1979
Cynomolgus monkeys	To confront protection of different vaccines.	Aerosol	Equivalent protection both with the vaccine currently employed in Australia (whole *C. burnetii* cell inactivated by formalin) and the vaccine obtained from chloroform/methanol residue of *C. burnetii*	Waag et al., 2002

The nematode *Caenorhabditis elegans* is susceptible to *C. burnetii* phase II and it develops acute infection. The fruit fly *Drosophila melanogaster* is susceptible to *C. burnetii* phase II; the infection can be induced by injection and it can cause mortality, which in females is dose-dependent (Bastos et al., 2017). Larvae of the greater wax moth, *Galleria mellonella*, have been used as a model of *C. burnetii* infection. *G. mellonella* can be maintained at 37°C and are simple to be manipulated and cheap. Moreover, they present several functional homologues of innate immune effectors in mammals (Bergin et al., 2005). This insect model has been used for different purposes such as to investigate virulence differences between *C. burnetii* NMI and NMII strains or to characterize of T4SS mutants (Norville et al., 2014), to identify virulence genes (Selim et al., 2018), or to map the transcriptome of *C. burnetii* during the infection (Kovacs-Simon et al., 2020).

In vertebrate models, such as mice, guinea pigs, and monkeys, the infection can be induced through aerosol inhalation, which is the natural route of transmission, or by intraperitoneal exposure. Susceptibility to the infection and signs experienced vary depending on species and strains (Bewley, 2013; Metters et al., 2019). Mice are often naturally resistant to the *C. burnetii* infection, but murine strains with specific immunologic alterations are available (Schoenlaub et al., 2016). For example, those with severe combined immunodeficiency (SCID) have been used to analyze the various components of immune response to the bacterium (Scott et al., 1978; Andoh et al., 2005). Ticks are also susceptible to *C. burnetii* infection with a possible role in the epidemiology of Q fever (Herrin et al., 2011; Chaligiannis et al., 2018; Varela-Castro et al., 2018).

Guinea pigs have been employed as models of human Q fever. These animals are more susceptible than mice and they develop high fever and splenic, hepatic, and bone marrow granulomas. In addition, they show blood abnormalities (as increased AST) and severe weight loss. In various studies, guinea pigs have been used to evaluate the histopathological alterations induced by *C. burnetii* infection after intraperitoneal or aerosol exposure (Heggers et al., 1975). Similar experiments have been conducted on murine models, and it was observed that, in mice, lesions were close to those seen in guinea pigs but more diffused (Scott et al., 1978).

As regards larger animals, nonhuman primates are particularly interesting as a model of the course of human Q fever (Gonder et al., 1979). Because of these characteristics, nonhuman primates can be useful to understand the disease caused by *C. burnetii* in humans and to develop a more reliable vaccine. However, monkeys are less employed as experimental models than mice and guinea pigs because of farming and use costs, space required, and ethical problems raised. Finally, *in vitro* and *ex vivo* models deserve to be cited as experimental models. They consist respectively in single cells (e.g., alveolar macrophages) and in perfused organ or tissue, and they have been employed to study the *C. burnetii* biology and its interaction with host at the cellular and tissue level. These models have been essential to comprehend *C. burnetii* intracellular replication and the host cell response to the infection (Voth and Heinzen, 2007; van Schaik et al., 2013). Other animal species (e.g., dogs, lions, and camels; Torina et al., 2007; Hornok et al., 2013; El Tigani-Asil et al., 2021) are susceptible to *C. burnetii* but have not been used as experimental models. *Coxiella-*related tick endosymbionts have also been identified as microbiota component of some tick species (Díaz-Sánchez et al., 2019).

As concerning molecular studies, a recent study aimed at characterization of the transcriptome of murine alveolar macrophages infected with a *Coxiella* strain mutant for type IVB secretion system (T4BSS) effector identified a set of inflammatory genes significantly upregulated in T4BSS mutant-infected cells compared to controls, suggesting a downregulating role of *Coxiella* T4BSS effector proteins for these genes. In particular, IL-17 signaling pathway was significantly affected with the T4BSS mutants that exhibited significantly more sensitivity to IL-17 than WT bacteria.

An increased expression of IL-17 downstream signaling genes, including the proinflammatory cytokine genes Il1a, Il1b, and Tnfa, the chemokine genes Cxcl2 and Ccl5, and the antimicrobial protein gene Lcn2, resulted in T4BSS mutant-infected cells compared to controls, confirming that *Coxiella* downregulates IL-17 signaling in a T4BSS-dependent manner in order to escape the macrophage immune response (Clemente et al., 2018).

Another study was aimed to discover new effector proteins involved in *C. burnetii* innate immune evasion process by the Dot/Icm T4SS. In particular, the authors found a new *C. burnetii* effector protein, NopA (nucleolar protein A), which interacts with Ras-related nuclear protein (Ran). Since it localizes at nucleoli of infected cells, NopA triggers the accumulation of Ran-GTP at nucleoli, thus perturbing the nuclear import of transcription factors of the innate immune signaling pathway. Cells exposed to *C. burnetii* strains mutant for NopA or for the Dot/Icm system presented a functional innate immune response, unlike cells exposed to wild-type *C. burnetii* or to a nopA complemented strain, confirming the role of NopA as *C. burnetii* regulator of the host innate immune response (Burette et al., 2020).

A comparative microarray-based study found that *C. burnetii* protein synthesis modulated (≥2 fold change) 36 host cell genes, codifying proteins principally involved in innate immune response, cell death and proliferation, vesicle trafficking and development, lipid homeostasis, and cytoskeletal organization, as suggested by the ontological analysis. The authors suggested that the production of *C. burnetii* proteins modulates the host cell gene expression in order to avoid the immune response, promote the host cell survival, and direct the development and maintenance of a replicative parasitophorous vacuole by controlling vesicle formation and trafficking in the host cell (Mahapatra et al., 2010).

## Current Diagnostic Approaches for *Coxiella burnetii*

Traditionally, laboratory diagnosis of *C. burnetii* can be carried out by detecting bacterial DNA (direct methods) or specific antibodies (indirect methods) in blood samples (Natale et al., 2012; Sahu et al., 2020).

Bacterium isolation is not a recommended diagnostic method since *C. burnetii* is classified as a level 3 containment microorganism and it requires biosafety level 3 conditions and prolonged incubation period (Sahu et al., 2020). Direct isolation can be obtained by inoculation of embryonated chicken eggs or cell culture, for example, in Vero cells or in L929 mouse fibroblasts. With heavily multi-contaminated samples, the inoculation of laboratory animals may be required and guinea pigs and mice are considered the most appropriate laboratory animals for this purpose (OIE, 2018). Also, a cell-free or axenic medium has been developed for *C.burnetii* culture, known as acidified citrate cysteine medium 2 (ACCM-2) (Omsland et al., 2009). The medium reduces oxidative stress (Sandoz et al., 2014) and mimics the intracellular environment of the host, allowing the propagation of the bacteria in both broth and solid agarose-based medium, at 37°C in a 2.5% O_2_ and 5% CO_2_ environment (Omsland et al., 2011). Another isolation medium, known as ACCM-D, has been formulated, including a supplementation of arginine, which further reduces the oxidative stress (Dresler et al., 2019). Axenic cultivation of *C. burnetii* will improve studies on its pathogenesis and genetics and will allow genetic manipulation of the pathogen (Omsland et al., 2009).

*C. burnetii* in tissue sections can be observed following staining with hematoxylin-eosin, periodic acid-schiff base, gomori methylamine silver stain, Giemsa, Warthin starry, and Gimenez technique (Woc-Colburn et al., 2008).

Microscopy-based observation can be carried out and electron microscopy can be used for pathogenicity studies, but it requires well equipped laboratory and skilled personnel and it is also a very expensive method (Justis et al., 2017). Other microscopic examinations include fluorescent microscopy, confocal microscopy, immunofluorescence, and/or immunoelectron microscopy, which can be used, respectively, to analyze intracellular growth and pathogenicity (Howe et al., 2003a; Howe et al., 2003b), to monitor phagolysosomal vacuole formation, localization, and multiplication of the microbes inside the CCV (Desnues et al., 2009; Miller et al., 2019; Moormeier et al., 2019), and to assess the location of a specific antigen or protein inside the bacterium (Morgan et al., 2010).

The polymerase chain reaction (PCR), an example of direct test, is particularly sensitive in the early stage of the infection, but its sensitivity decreases quickly following specific antibiotic treatment. Nowadays, *C. burnetii* infection is confirmed by real-time PCR detection, within 2 weeks after acute infections in sero-negative individuals together with clinical data and diagnostic imaging for the diagnosis of chronic Q fever (Schneeberger et al., 2010; Kampschreur et al., 2015; Eldin et al., 2017). The insertion sequence *IS 1111* gene is considered as the target of choice in PCR detection of *C. burnetii*, as the repetitive element has a multi-copy gene that increases sensitivity of the method (Klee et al., 2006). Loop-mediated isothermal amplification (LAMP-PCR) assay presents with additional advantages such as rapidity, visual interpretation of results, and the possibility to carry out the reaction at a temperature between 60 and 65°C (Das et al., 2018).

Multi-spacer sequence typing (MST) and multi-locus variable number tandem repeat analysis (MLVA) are used for pathogen genotyping of *C. burnetii* (Galiero et al., 2016; Dabaja et al., 2020).

Immunological assays are useful once antibodies are detectable in the patient serum, about 10 days after the beginning of the disease, and the presence of antibodies against *Coxiella* in the serum still represent the gold standard for the diagnosis of the infection or past exposure to the Gram-negative coccobacillus. In the specific, complement fixation test was the previous reference test reported by OIE, but it was replaced due to its low sensitivity (OIE, 2018). The immunofluorescence assay (IFA) is more reliable even a year after acute infection or in chronic Q fever than other serological assays, such as ELISA or complement fixation test (Wegdam-Blans et al., 2012; Wegdam-Blans et al., 2014), although the absence of antibodies measured by IFA does not exclude a past exposure. Moreover, a different immune response is revealed in the presence of acute or chronic infection; as in acute Q fever, antibodies against *C. burnetii* phase II are prevalent, whereas in chronic Q fever, antibodies against *C. burnetii* phase I are the most represented (Mori et al., 2017).

Commercial ELISA kits able to detect both phase I and phase II stage antibodies or phase-specific antibodies are available (Kantsø et al., 2012; Meekelenkamp et al., 2012; Wegdam-Blans et al., 2014; El-Mahallawy et al., 2016) and ELISA represents a rapid, highly sensitive, and specific diagnostic assay (OIE, 2018).

In Australia, serology is also accompanied by a skin test, similar to tuberculin skin test, for evaluating cell-mediated response to *C. burnetii* (Marmion et al., 1990; Ruiz and Wolfe, 2014), but often the results coming from both assays are discordant. For this reason, other more sensitive and specific assays are under investigation.

Recently, it has been reported that *C. burnetii*-specific T-cell IFN-γ release may represent a sensitive and long-lasting marker of past *C. burnetii* exposure (Scholzen et al., 2021). The evaluation of cell-mediated responses to *Coxiella* through IFN-γ release assay (IGRA) could be very useful to detect past infections, since cellular response persists also in sero- and skin test-negative individuals (Schoffelen et al., 2014a; Schoffelen et al., 2013; Scholzen et al., 2021). It is still unknown which fraction of *C. burnetii* is responsible for IFN-γ release, but reported data show the existence of some immunodominant-specific peptides from its proteins that are recognized in IGRA-positive when compared with IGRA-negative subjects. Some endotoxins can also induce IFN-γ release from T and NK cells, probably mediated by monocytes, activated by *Coxiella* LPS, endothelial cells, and other cytokines (Le et al., 1986; Tennenberg and Weller, 1996; Lauw et al., 2000). Among T cells, preliminary data (Scholzen et al., 2021) indicate CD4^+^ cells as the major source of IFN-γ, followed by CD8^+^ T cells.

## Protection of *Coxiella burnetii* Infected Mice Was Increased by TLR Triagonists Used as Adjuvants

**Figure 2:**
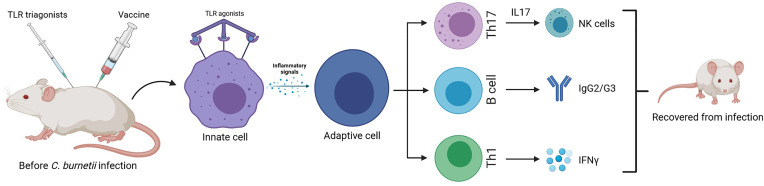
Murine model of enhanced protective response against *C. burnetti* infection: vaccines with TLR triagonists as adjuvants, activating multiple Toll-like receptors simultaneously, induce a strong antigen-specific humoral and cell-mediated (Th1) immune response against *Coxiella* infection, being a more successful vaccination strategy than the traditional one (Gilkes et al., 2020).

Researchers observed in *C. burnetii* immunized mice a new successful component for vaccination strategies that takes advantage of a typical innate immunity receptor: TLR triagonists as good adjuvants to increase protection from infection symptoms (Gilkes et al., 2020). Using these new types of adjuvants that are able to stimulate immunity through binding simultaneously three different TLRs, the *C. burnetii*-specific Th1 and Ig responses were potentiated. Mice previously infected and treated with different TLR tritagonists do not suffer the typical symptoms of *C. burnetii* infections (e.g., fever, weight loss). These protective effects exerted by TLR tritagonists were accompanied by a beneficial *Coxiella*-specific adaptive immunity. In fact, mice receiving TLR tritagonists developed strong antigen-specific IgG and Th1 cells able to sustain a *Coxiella*-specific immune response (**Figure 2**).

In addition, TLR stimulation of dendritic cells and other antigen-presenting cells represents a strong proinflammatory signal capable of overcoming even the control of regulatory T cells (Tregs). In fact, TLR expression has been reported also in adaptive immune lymphocytes, suggesting that the interaction with TLR ligands could stimulate or inhibit these cells (Hisaeda et al., 2004).

Further studies are needed to investigate the role of other cells potentially able to be involved in TLR triagonist effects such as Tregs, whose activation could be helpful for both host and pathogen, and innate lymphoid cells (ILCs) that could exert protection topically in the lungs and/or systemically. Another important effect necessary to better understand TLR tritagonist protections is represented by the analysis of T- and/or B-cell memory after the injection of TLR triagonists.

## Conclusions

Non-human experimental models of protection are needed as a preclinical stage for the development of effective vaccination strategies. Taken together, a link between innate and adaptive immunity seems to be a good model to induce protection against *C. burnetii* infection, even if LPS detrimental effects are detected. Avoiding these side effects to design pre-clinical and clinical vaccination strategies could be a goal of future research in these fields. Definition of the protection exerted by other effector mechanisms such as ILCs or Th17-induced neutrophilia is necessary to complete the immunological scenario that supports protection against *C. burnetii* in TLR tritagonist animals.

An effort to define the effects of TLR triagonists on antigen-specific T- and B-cell memory could give a substantial completion of protection against *C. burnetii* infection (Fratzke et al., 2021). Furthermore, to translate this vaccination strategy using these adjuvants needs a complete definition of multiple immunological scenarios. Additionally, new immunodiagnostic assays, such as IGRA, could become more appreciable tools to diagnose past *C. burnetii* infections and they may result very helpful especially in the context of pre-vaccination screens. The application of latest omics, genetic, and *in silico* technologies for the identification of conserved T-cell epitopes in multiple host species, the development of genetically modified organisms, and the design of multi-epitope antigens are promising approaches for collaborative integration in the development of more effective and safe vaccines against Q fever (Gerlach et al., 2017; Reeves et al., 2017; Jaydari et al., 2020; Long et al., 2021; Nooroong et al., 2021; Piel et al., 2021).

## Author Contributions

Conceptualization and design: GS and VB. Supervision: AT. Funding acquisition: AT and AG. Drafting the article: VB, LP, FG, and DL. Software: GB. Revision of article: GS and JF. All authors contributed to the article and approved the submitted version.

## Funding

This research was funded by the Italian Ministry of Health with grants RC IZSSI 08/19 and RC IZSSI 01/20.

## Conflict of Interest

The authors declare that the research was conducted in the absence of any commercial or financial relationships that could be construed as a potential conflict of interest.

## Publisher’s Note

All claims expressed in this article are solely those of the authors and do not necessarily represent those of their affiliated organizations, or those of the publisher, the editors and the reviewers. Any product that may be evaluated in this article, or claim that may be made by its manufacturer, is not guaranteed or endorsed by the publisher.
